# Elucidation of Baicalein’s Anti-Intervertebral Disc Degeneration Mechanism Through Network Pharmacology and Experimental Validation

**DOI:** 10.1007/s12013-026-02146-5

**Published:** 2026-08-24

**Authors:** Rui Wang, Zhiyong Liu, Xiaopeng Zhao

**Affiliations:** 1 Department of Orthopedic Trauma, Shandong Second Provincial General Hospital, Jinan, 250022 China; 2 Department of Spine Surgery, Shandong Second Provincial General Hospital, Jinan, 250022 China

**Keywords:** Baicalein, Intervertebral disc degeneration, Extracellular matrix metabolism, mitogen-activated protein kinase signaling

## Abstract

This study aims to elucidate the biological effects of Baicalein (BA) on intervertebral disc degeneration (IDD) and the potential mechanisms involved. Network pharmacology analysis was performed to identify the core targets and signaling pathways responsible for the therapeutic effects of BA on IDD. In vitro validation was carried out using IL-1β-stimulated nucleus pulposus cells (NPCs). Cell viability, apoptosis, inflammatory cytokines (TNF-α, IL-6), extracellular matrix (ECM)-related molecules (MMP3, Aggrecan, Collagen II), and the phosphorylation of ERK and p38 were measured using CCK-8 assay, flow cytometry, qRT-PCR, ELISA and Western blot. Network pharmacology identified 80 common targets and highlighted the MAPK signaling pathway as a key mechanism. In vitro, BA (50 µmol/L) mitigated IL-1β-induced cytotoxicity in NPCs and promoted cell proliferation. BA significantly inhibited apoptosis by regulating the Bcl-2/Bax family and cleaved c-caspase-3. It also decreased the expression of TNF-α and IL-6, and reversed ECM homeostasis by modulating MMP-3, Aggrecan and Collagen II expression. Furthermore, BA treatment effectively suppressed IL-1β-induced phosphorylation of ERK and p38. Notably, the MAPK agonist anisomycin partially abolished the above protective phenotypes of BA and restored MAPK phosphorylation. BA alleviates apoptosis, inflammatory response and ECM degradation in NPCs, to retard IDD development mainly through inhibiting MAPK pathway activation, supporting the clinical translational potential of BA for IDD treatment.

**Supplementary Information:**

The online version contains supplementary material available at https://doi.org/10.1007/s12013-026-02146-5.

## Introduction

Intervertebral disc degeneration (IDD) is recognized as the primary pathological basis of low back pain and radicular symptoms, imposing a substantial burden on the global healthcare system and socioeconomic resources [1, 2]. This condition is fundamentally characterized by a progressive deterioration, mainly involving inflammation, cell injury and abnormal extracellular matrix (ECM) metabolism, along with nucleus pulposus cells (NPCs) senescence and apoptosis [3, 4]. Given the limited effectiveness of current treatments in altering the disease progression, it is critical to develop safe, potent drugs targeting the key pathological mechanisms in IDD.

**Fig. 1:**
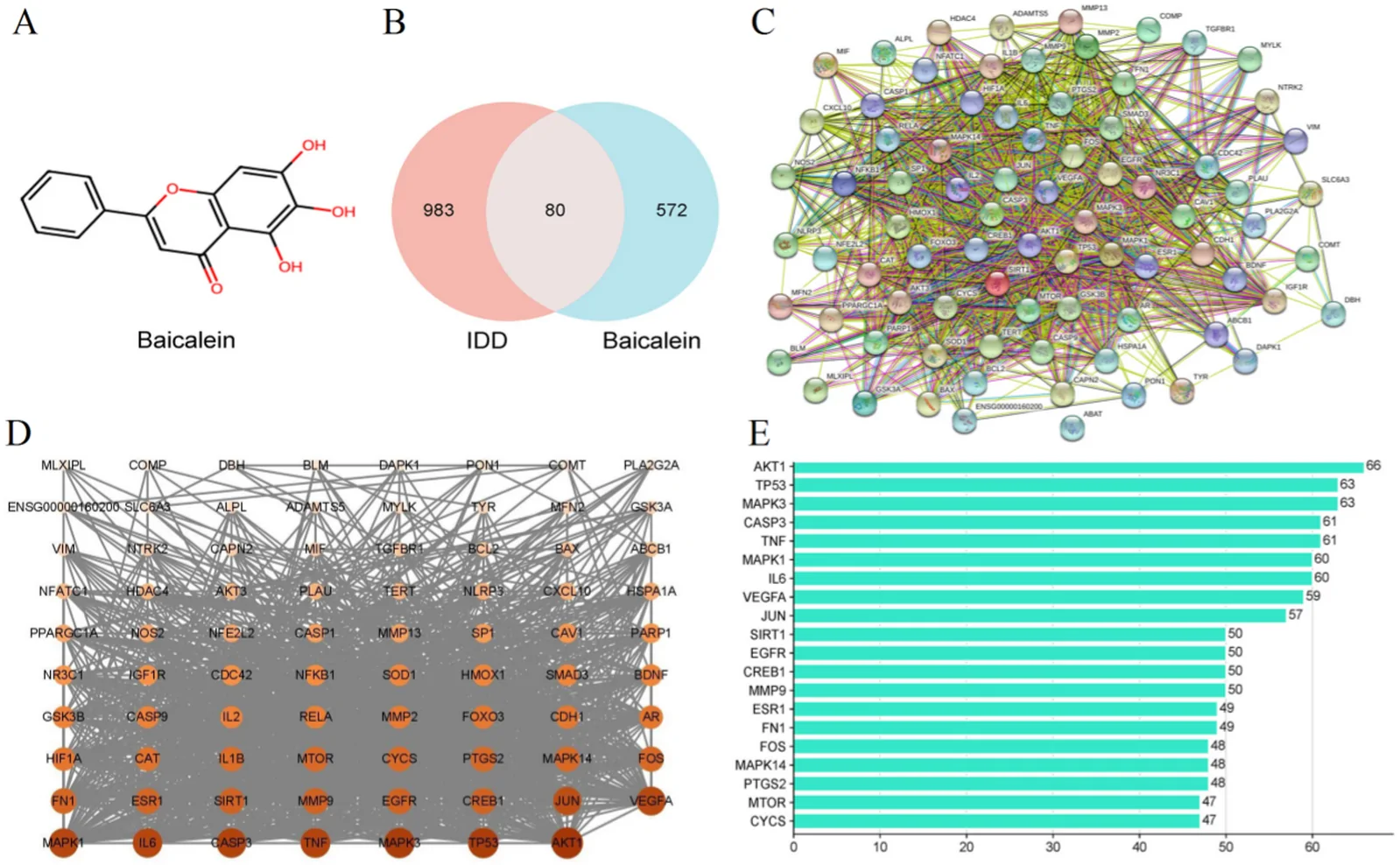
PPI network associated with BA intervention for IDD. (**A**) Chemical structure of BA. (**B**) Venn diagram-communicative targets. (**C**) STRING-based PPI network. (**D**) PPI network mapped in Cytoscape. (**E**) Degree value of the intersectional target

In recent years, the exploration of bioactive natural products with multi-target regulatory potential has emerged as a promising strategy for developing interventions against chronic degenerative diseases. Baicalein (BA, Fig. 1A), a natural flavonoid extracted from the rhizome of *Scutellaria baicalensis*, is a classic Chinese herbal medicine. It possesses diverse biological properties, covering anti-inflammation, antioxidation, anti-apoptosis, and mitochondrial protection [5, 6]. Mechanistically, it has been shown to suppress classical inflammatory cascades, including nuclear factor-κB (NF-κB) and mitogen-activated protein kinase (MAPK) pathways, thereby downregulating the release of interleukin-1β (IL-1β) and tumor necrosis factor-α (TNF-α) [5, 7, 8]. Given its capacity to restrain chondrocyte apoptosis and slow joint degenerative changes, BA has drawn extensive research attention for osteoarthritis intervention [9]. Meanwhile, current research has found that BA can attenuate IDD. Nevertheless, whether BA can effectively mitigate IDD, and through which specific targets and downstream signaling networks it acts, remains to be systematically elucidated.

As an innovative cross-disciplinary method combining systems biology, bioinformatics and computational pharmacology, Network pharmacology offers a multidimensional “disease–gene–target–drug” analytical framework [10]. This approach is especially well-suited for clarifying the mechanisms of action of natural compounds, which typically display multi-target properties in complex disease contexts [11]. By constructing compound-target interaction networks and mapping them to disease-relevant gene sets, network pharmacology allows for the systematic identification of core therapeutic targets and key signaling pathways. These predictions can then be formulated into testable hypotheses and guide subsequent experimental validation.

This study integrated network pharmacology prediction and cellular experiments to systematically explore the pharmacological mechanism of BA against IDD. This work aims to uncover potential targets and pathways through which BA exerts its anti-degenerative effects, thereby providing mechanistic insights and a theoretical foundation for the further development of BA-based therapies, as well as offering new perspectives for the utilization of traditional Chinese medicine in degenerative disc diseases.

## Materials and Methods

### Network Pharmacology Methods

#### Obtaining Gene Targets for BA and IDD

The SMILES structural formula of BA was obtained using the PubChem database (https://pubchem.ncbi.nlm.nih.gov/). Then import BA’s SMILES structure into the Swiss Target Prediction database (http://www.swisstargetprediction.ch), targetnet database (http://targetnet.scbdd.com/home/targets/) and Similarity Ensemble Approach database (SEA, http://sea.bkslab.org/), to obtain BA targets. Using “baicalein” as the search term, the BA-related targets were retrieved respectively in the Traditional Chinese Medicine Systems Pharmacology database (TCMSP, https://tcmsp-e.com/) and the HERB database (http://47.92.70.12/).

IDD targets were obtained by searching the Online Mendelian Inheritance in Man (OMIM, https://www.omim.org/), Gene Cards database (https://www.genecards.org/), DisGeNET (https://disgenet.com/), and related literature with the search term “Intervertebral disc degeneration”, and were then merged and de-duplicated. Among them, GeneCards uses the median of relevance score > = this value as the screening criterion to obtain therapeutic targets.

Gene information was standardized in the UniProt database (https://www.uniprot.org/). Target intersection and union analyses were performed using Venny (https://bioinfogp.cnb.csic.es/tools/venny/index.html). After excluding redundant targets, the union set of targets associated with BA and IDD was identified.

#### Protein-Protein Interaction Data

The overlapping targets were imported into the STRING database (https://cn.string-db.org/), with species set to “Homo sapiens”, analysis mode as “Multiple proteins”, and interaction confidence score was set as 0.4. After removing isolated nodes, the resulting data was loaded into Cytoscape 3.9.0 for visual analysis, constructing a protein-protein interaction (PPI) network and generating a degree distribution histogram of core targets.

#### Analysis of Biological Functions and Pathways

Functional enrichment of the predicted targets was carried out via Gene Ontology (GO) enrichment analysis and Kyoto Encyclopedia of Genes and Genomes (KEGG) analyses using the Metascape platform. With a threshold of *p* < 0.05, significantly enriched GO items [including biological process (BP), cellular component (CC) and molecular function (MF)] and KEGG pathways were identified. The results were further visualized using the online bioinformatics software.

### In Vitro Experimental Verification

#### Experimental Cells and Reagents

Healthy human primary NPCs were obtained from Wuhan Shangen Biotechnology Co., Ltd. BA (purity > 98%) and IL-1β were purchased from Beyotime Biotechnology Co., Ltd. All antibodies were sourced from Wuhan Sanying Biotechnology Co., Ltd. and Abcam (UK). Other reagents used in this study were acquired from Beijing Solarbio Technology Co., Ltd.

#### Cell Culture

Human primary NPCs were cultured in MEM/F12 medium containing 10% fetal bovine serum. Cells were incubated at 37℃ under 5% CO_2_ humidified conditions. Cellular growth status was routinely observed microscopically. Cells were subcultured when reaching 80% confluence. For all experiments, cells from the 3rd to 4th passages were used.

#### Processing of NPCs

NPCs were allocated into different groups for a series of experiments. First, to determine the optimal intervention concentration of BA, a preliminary concentration screening was performed: cells were incubated with BA at gradient concentrations of 0, 5, 25, 50, 75 and 100 µmol/L. Based on the outcomes of these initial experiments, the selected optimal concentrations of BA were applied in the subsequent experimental design. Accordingly, NPCs were assigned into four groups: blank control (BC) group, IL-1β group (Cells were treated with 10 ng/mL IL-1β), BA + IL-1β group (Cells were treated with the optimal concentration of BA for 2 h before 10 ng/mL IL-1β stimulation), BA + IL-1β + anisomycin group (Cells were treated with the optimal concentration of BA for 2 h before 10 ng/mL IL-1β and 2 µg/L anisomycin treatment).

#### CCK-8 Assay

NPCs were inoculated into 96-well plates at 6 × 10^3^ cells per well and cultured for 24 h to allow attachment. Following this initial adherence period, the cells were processed in line with the predefined experimental groups and incubated for an additional 24 h. Subsequently, 10 µL of CCK-8 reagent was supplemented and incubated for 2 h. Cell viability was quantified by measuring the absorbance value at 450 nm.

#### Apoptosis Assay

NPCs were seeded in 96-well plates, digested with trypsin upon reaching appropriate confluence, and rinsed twice with PBS. Collected cells were resuspended in 500 µL binding buffer, then stained with 5 µL Annexin V-FITC and 5 µL PI. The staining reaction lasted 15 min at room temperature away from light. Apoptosis rates were subsequently analyzed using a flow cytometer.

#### Western Blot

Cellular total protein was isolated via RIPA lysis buffer, and protein concentrations were determined with a microplate reader. Samples underwent electrophoresis on 10% SDS-PAGE gel, then electrotransferred onto 0.45 μm PVDF membranes. Membranes were sealed with 5% skim milk at room temperature for 2 h. Afterwards, they were incubated overnight at 4℃ with the following primary antibodies at 1:1000 dilution, including Bax (50599-2-Ig), c-caspase-3 (68773-1-Ig), Bcl-2 (12789-1-AP), Collagen II (86139-2-RR), Aggrecan (68350-1-Ig), MMP-3 (17873-1-AP), ERK (11257-1-AP), p-ERK (28733-1-AP), p38 (14064-1-AP), p-p38 (28796-1-AP) and GAPDH (60004-1-Ig). Following washing, HRP-labeled secondary antibodies were applied and incubated at 37℃ for 2 h. Protein bands were developed with ECL reagent and detected with an ECL imaging system. Band gray values were analyzed by Image J software, with GAPDH serving as the internal reference.

#### qRT‒PCR Analysis

Cellular total RNA was isolated with TRIzol reagent, and its concentration and purity were measured. cDNA was synthesized via a reverse transcription kit. qPCR amplification was carried out using a commercial PCR kit, with cDNA as the template, along with gene-specific primers and dNTPs. Each sample was tested in three replicates. β-actin acted as an internal reference. The relative expression of TNF-α, IL-6, MMP-3, aggrecan and collagen II was calculated adopting the 2^−ΔΔCt^ approach. Primer sequences employed in this experiment are provided in Table 1.

**Table 1:** Primer sequences used in qRT-PCR

Gene	Forward (5’→3’)	Reverse (5’→3’)
TNF-α	CAGCCTCTTCTCCTTCCTGAT	GCCAGAGGGCTGATTAGAGA
IL-6	AGACAGCCACTCACCTCTTCA	GGCTTGTTCCTCACTACTCTC
MMP-3	CCCTGCAACCGTGAAGAAGA	GACAGCATCCACCCTTGAGT
Aggrecan	TGAGCGGCAGCACTTTGAC	TGAGTACAGGAGGCTTGAGG
Collagen II	TGGACGATCAGGCGAAACC	GCTGCGGATGCTCTCAATCT
β-actin	CCCTGGAGAAGAGCTACGAG	CGTACAGGTCTTTGCGGATG

#### ELISA

The contents of TNF-α and IL-6 in NPCs were determined using commercial ELISA kits (SEKH-0047 and SEKH-0013), and all experimental procedures were conducted following the manufacturer’s protocols.

### Statistical Analysis

GraphPad Prism 8.0 was used for statistical processing. The normality of data distribution and homogeneity of variance were tested first. Experimental data were expressed as mean ± SD. One-way ANOVA was performed for group comparisons, and Tukey’s post-hoc test was used for multiple pairwise comparisons. All experiments were conducted with three independent biological replicates. *P* < 0.05 was regarded as statistically significant.

## Results

### Intersectional Targets of Diseases and Drugs and Building Protein Interaction Networks

This study employed multiple databases to screen targets associated with BA and IDD. The network pharmacology was adopted to explore the potential mechanism by which BA alleviates IDD. By utilizing target prediction websites, a total of 652 BA-related targets and 1063 IDD-related targets were obtained. Venny 2.1.0 was used to plot the Venn diagram, revealing 80 overlapping genes between BA and IDD (Fig. 1B). A PPI network was built using the STRING database, containing 79 nodes and 1237 edges (Fig. 1C). After excluding isolated nodes, the network was visualized with Cytoscape 3.9.0 (Fig. 1D). Degree analysis, demonstrated that AKT1 (degree = 66), TP53 (degree = 63), MAPK3 (degree = 63), CASP3 (degree = 61) and TNF (degree = 61) exhibited high interaction frequency (Fig. 1E), These core genes are speculated to be critical for the therapeutic effect of BA against IDD.

### Functional and Pathway Enrichment Analysis

**Fig. 2:**
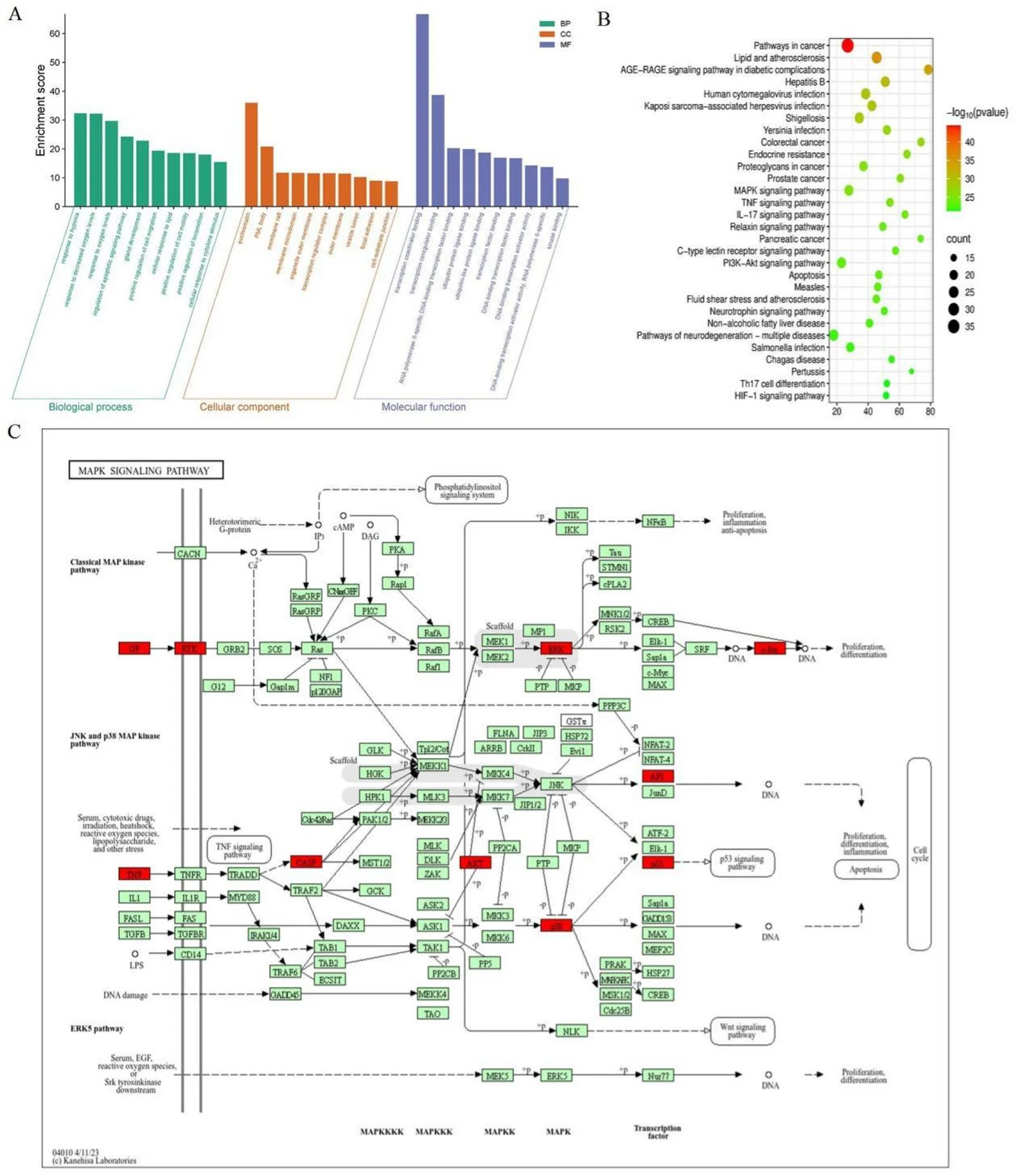
Functional enrichment of putative targets via GO and KEGG analysis. (**A**) The top 10 BP, CC, and MF terms ranked by *p*-value from GO enrichment. (**B**) KEGG enrichment bubble chart of BA targets for IDD treatment. (**C**) Schematic view of MAPK pathways; key segments are labeled in red boxes, indicating dominant genes and pathways responsible for BA bioactivity (KEGG Mapper Color)

Shared drugs-diseases were submitted to the Metascape database for GO and KEGG pathways enrichment analyses. 1444 BP, 66 CC, 114 MF of GO functions and 173 KEGG pathways were obtained. The top 10 GO terms ranked by *P*-value were selected for graphical presentation (Fig. 2A). The dominant BP terms involved hypoxia, response to decreased oxygen levels, regulation of apoptotic signaling pathway, etc. The CC terms mainly include euchromatin, PML body, membrane raft, membrane microdomain, etc. The MF terms mainly include transcription coactivator binding, transcription coregulator binding, RNA polymerase II-specific DNA-binding transcription factor binding, etc. These findings revealed that BA exerts therapeutic effects on IDD via diverse biological functions. The top enriched 30 KEGG pathways are displayed in Fig. 2B. Key pathways associated with BA treatment against IDD include the AGE-RAGE, MAPK, TNF, IL-17, PI3K-Akt signaling pathway, apoptosis, etc. Among them, the MAPK pathway showed a high ranking, implying its vital role in the anti-IDD action of BA. The detailed MAPK pathway is illustrated in Fig. 2C, where red rectangles mark the target genes responsible for BA’s pharmacological activity.

### BA Affects NPCs’ Viability

**Fig. 3:**
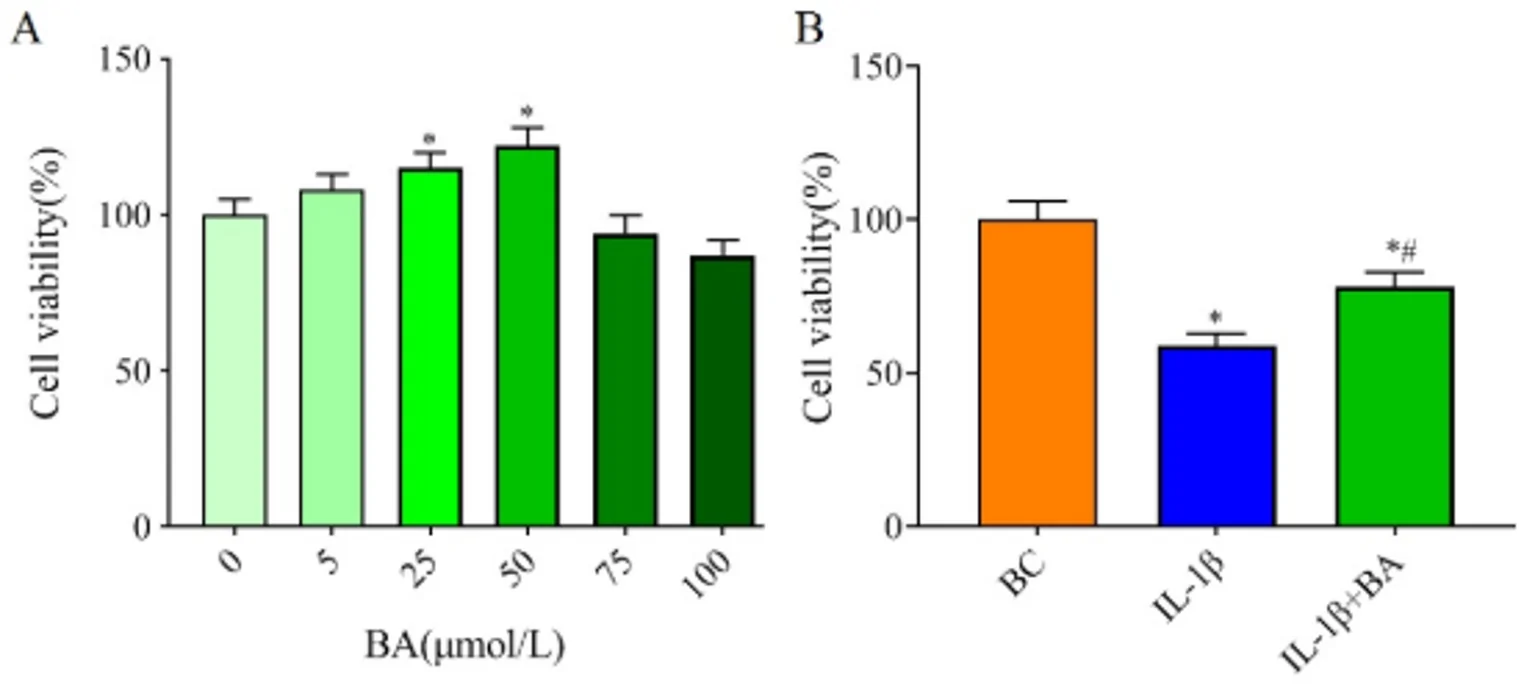
BA affects NPCs’ viability. (**A**) Cytotoxicity of BA at gradient concentrations tested by CCK-8 assay. (**B**) Changes in cell viability following BA intervention. ^*^P < 0.05, vs. BC group; ^#^*P* < 0.05, vs. IL-1β group

In this study, NPCs treated with BA at different concentrations (0, 5, 25, 50, 75 and 100 µmol/L). Cell viability was detected using the CCK-8 assay after 24 h of incubation. The results showed that cell viability gradually increased with the rise of BA concentration within the range of 0 to 50 µmol/L. The maximum cell viability was observed at 50 µmol/L, which was significantly different from the control group (*P* < 0.05). When the concentration exceeded 50 µmol/L, cell viability decreased gradually as the BA concentration further increased. Collectively, BA improved the viability of nucleus pulposus cells in a concentration-dependent manner, and 50 µmol/L was identified as the optimal concentration with the most potent effect (Fig. 3A). Therefore, 50 µmol/L BA was chosen for follow-up experiments. To simulate the degenerative microenvironment in vitro, an IDD model was established by stimulating NPCs with IL-1β. CCK-8 results confirmed that IL-1β markedly decreased NPCs’ viability, whereas BA effectively reversed this inhibitory effect (Fig. 3B).

### BA Regulates Apoptosis in IL-1β-Induced NPCs

**Fig. 4:**
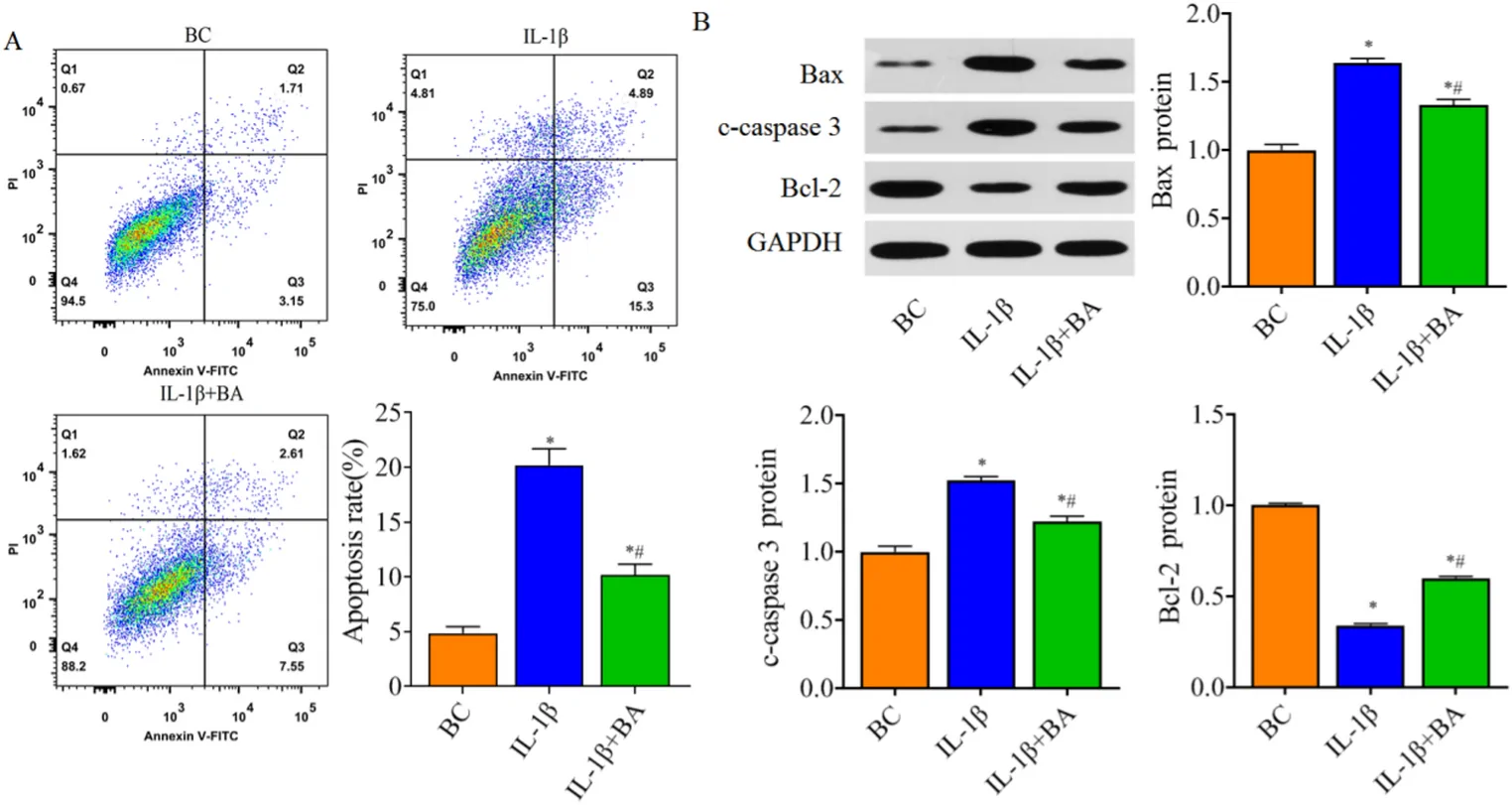
BA affects apoptosis in IL-1β-induced NPCs. (**A**) Apoptosis was examined by flow cytometry. (**B**) Levels of apoptotic proteins were analyzed by Western blot. ^*^P < 0.05, vs. BC group; ^#^*P* < 0.05, vs. IL-1β group

Excessive apoptosis of NPCs is a key driver of IDD development. Here, flow cytometry combined with Annexin V-FITC/PI assay staining was used to assess the influence of BA on cell apoptosis. As shown in Figs. 4A, The apoptosis rate significantly increased in NPCs following IL-1β stimulation (*P* < 0.05), whereas BA pretreatment effectively lowered this value. The Western blot detection results were further analyzed to elucidate the underlying molecular mechanism. As shown in Figs. 4B, relative to the BC group, IL-1β stimulation significantly increased the levels of pro-apoptotic proteins Bax and c-caspase-3, and decreased the expression of anti-apoptotic protein Bcl-2. Notably, BA intervention reversed the above protein changes. Taken together, BA can suppress apoptosis in IL-1β-induced NPCs.

### BA Alleviates Inflammation in IL-1β-Induced NPCs

**Fig. 5:**
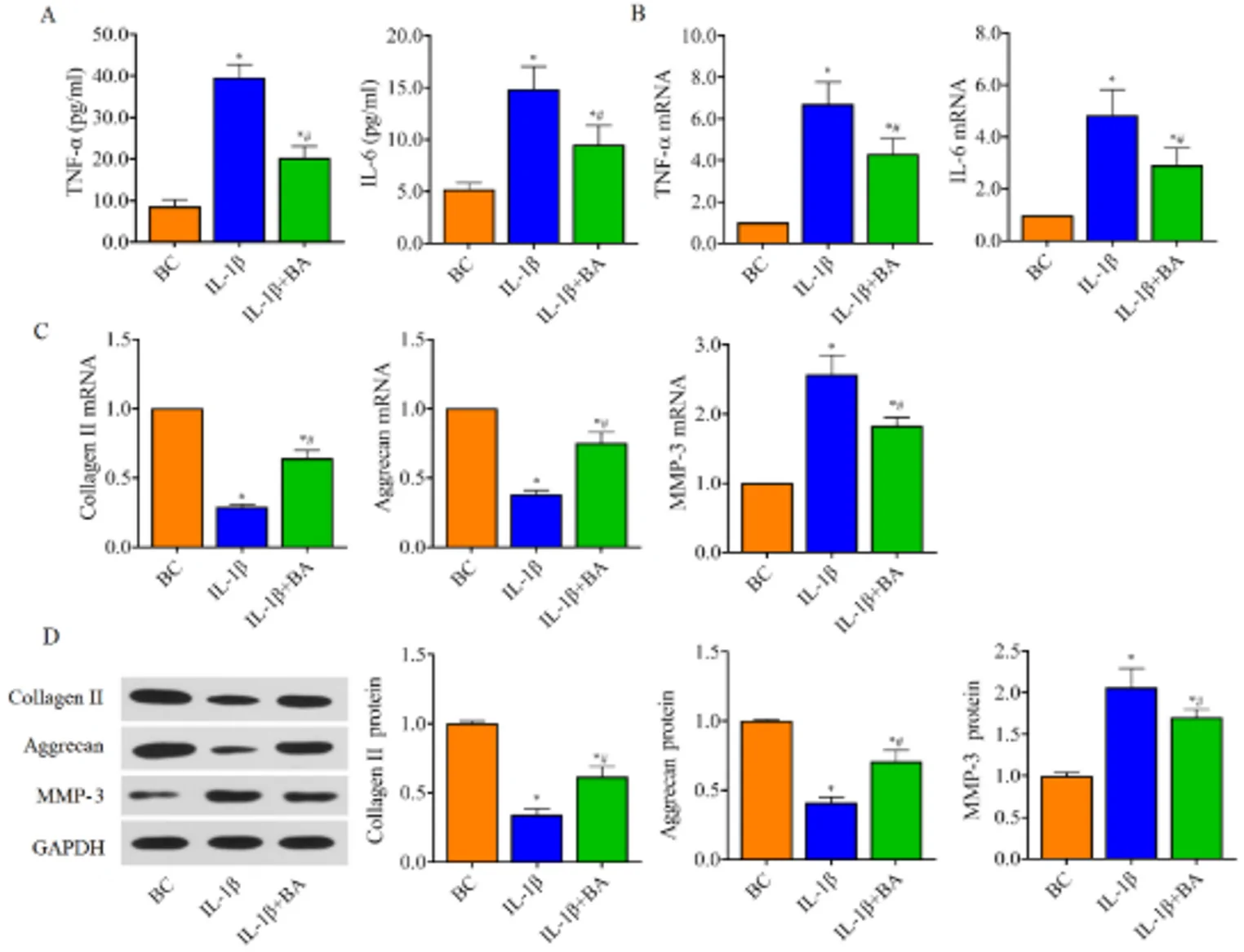
BA affects inflammation and ECM homeostasis in IL-1β-induced NPCs. (**A**, **B**) qRT-PCR and ELISA analyses for IL-6 and TNF-α expression. (**C**, **D**) qRT-PCR and Western blot detection of MMP3, Aggrecan and CollagenⅡ. ^*^P < 0.05, vs. BC group; ^#^*P* < 0.05, vs. IL-1β group

**Fig. 6:**
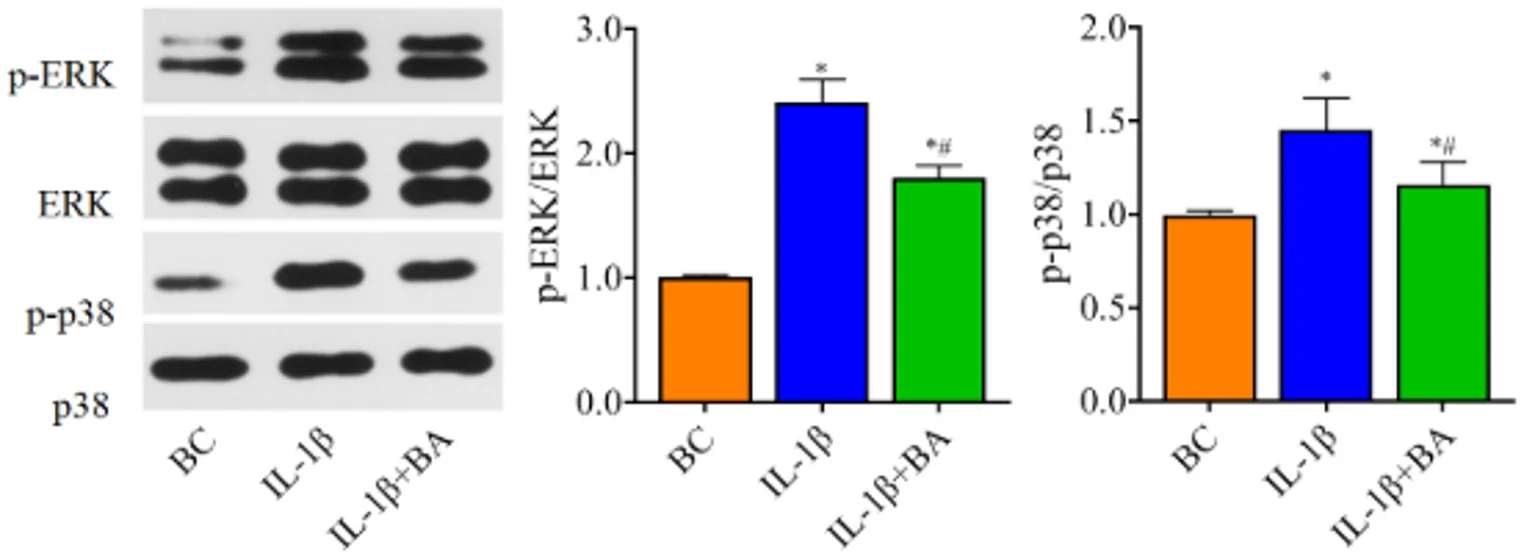
BA modulates the MAPK signaling pathway in IL-1β-induced NPCs. ^*^P < 0.05, vs. BC group; ^#^*P* < 0.05, vs. IL-1β group

Accumulating evidence suggests a close association between inflammation and IDD. Accordingly, the levels of the pro-inflammatory cytokines TNF-α and IL-6 were detected by qRT-PCR and ELISA, respectively. As shown in Figs. 5A and 6B, IL-1β stimulation significantly elevated the secretion of TNF-α and IL-6 compared to the control group. Conversely, BA intervention greatly inhibited such upregulation and reduced their contents. These findings suggest that BA attenuates the inflammatory response in IL-1β-induced NPCs.

### BA Modulates ECM Homeostasis in IL-1β-Induced NPCs

ECM stability relies on a dynamic balance between synthesis and degradation. The effect of BA on ECM metabolism was assessed by examining the expression of catabolic enzyme MMP-3 and the key structural components Aggrecan and Collagen II using qRT-PCR and Western blot analysis. As shown in Fig. 5C and D, IL-1β notably increased MMP-3 expression and downregulated Aggrecan and Collagen II expression. Conversely, BA treatment reversed these effects, decreasing MMP-3 expression and significantly increasing Aggrecan and Collagen II expression compared to the IL-1β group. Collectively, these results suggest that BA inhibits ECM catabolism and promotes the synthesis of its major structural components, thereby attenuating IL-1β-induced ECM degradation in NPCs.

### BA Modulates the MAPK Signaling Pathway in IL-1β-Induced NPCs

The MAPK cascade is pivotal for regulating inflammation and ECM metabolism during IDD progression. Its activation promotes inflammatory factor release, upregulation of matrix-degrading enzymes, and suppression of ECM synthesis. Accordingly, this study further explored whether BA affects MAPK pathway activation. As shown in Fig. 6, IL-1β dramatically activated the MAPK pathway in NPCs, as evidenced by increased phosphorylation of ERK and p38. This activation was effectively inhibited by BA treatment. These results indicate that BA suppresses the IL-1β-triggered MAPK signaling in NPCs.

### Anisomycin Rescue Experiment Confirms BA Targets the MAPK Pathway

**Fig. 7:**
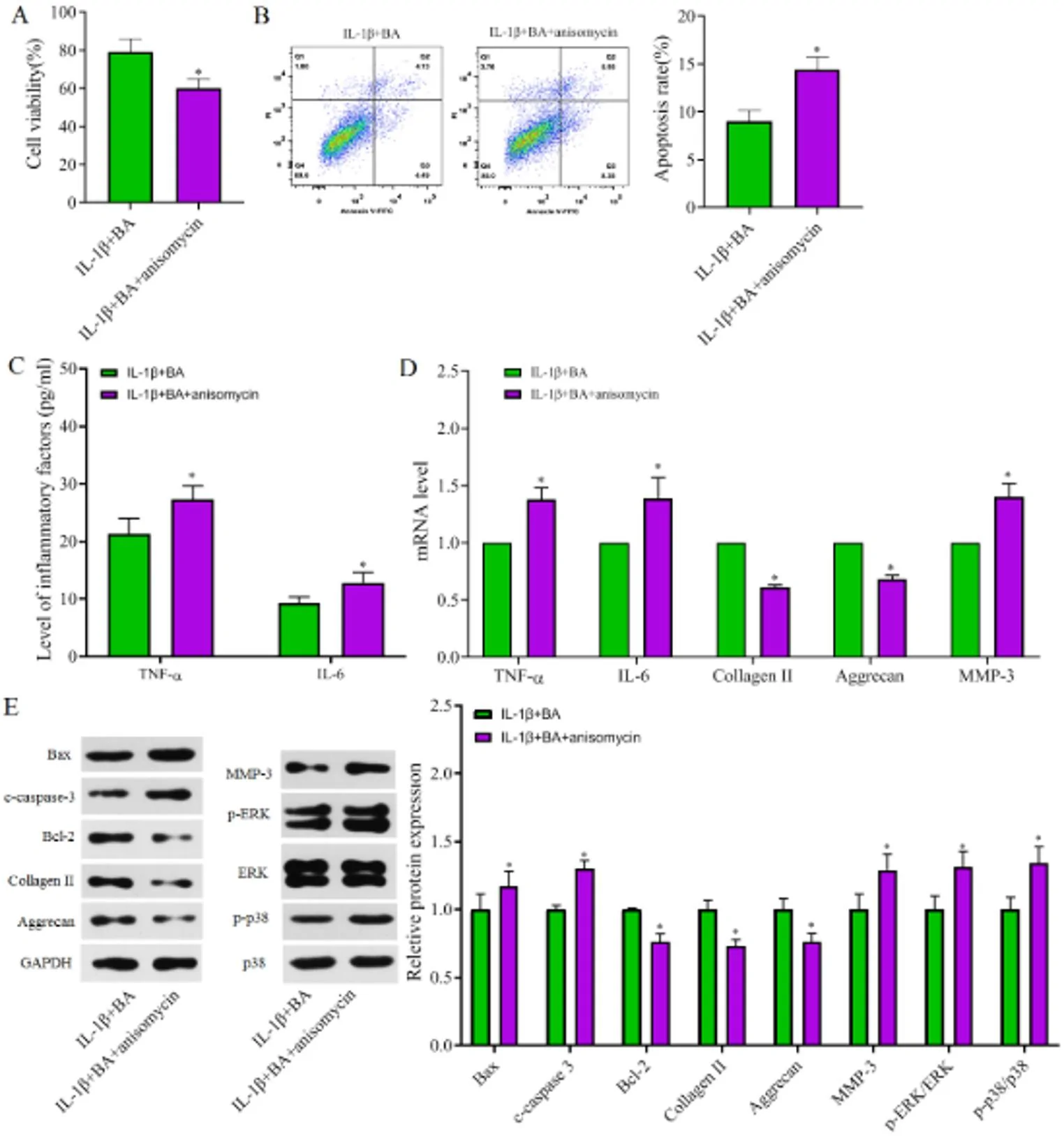
Rescue experiment with MAPK agonist anisomycin. (**A**) CCK-8 detection of cell viability. (**B**) Apoptosis was examined by flow cytometry. (**C**) ELISA measured concentrations of TNF-α and IL-6. (**D**) qRT-PCR analysis for mRNA levels of inflammatory and ECM-related genes. (**E**) Western blot detection of related proteins. ^*^P < 0.05, vs. IL-1β + BA group

MAPK agonist anisomycin was adopted for rescue experiments to verify that BA relies on MAPK to exert protection (Fig. 7). Compared with the IL-1β + BA group, anisomycin reversed BA’s protective function against IL1β-induced NPC injury. CCK-8 assays (Fig. 7A) showed that anisomycin reduced cell viability. Flow cytometry data (Fig. 7B) displayed markedly increased apoptotic rate. ELISA and qRT-PCR results (Fig. 7C, D) revealed that anisomycin elevated the expression of pro-inflammatory TNF-α, IL-6 and catabolic MMP-3, while inhibiting the mRNA levels of ECM anabolic Collagen II and Aggrecan. Western blot quantification (Fig. 7E) further validated the above molecular changes: anisomycin upregulated pro-apoptotic Bax, c-caspase-3 and MMP-3, downregulated anti-apoptotic Bcl-2, Collagen II and Aggrecan, and restored BA-suppressed phosphorylation of ERK and p38. Collectively, these rescue findings prove BA ameliorates IL-1β-triggered NPC damage via suppressing the MAPK signaling cascade.

## Discussion

IDD and its secondary pathological changes are the leading causes of low back pain (LBP), serving as the fundamental pathological basis for a series of spinal degenerative conditions such as spinal instability, cervical spondylosis, and lumbar spinal stenosis [12]. Although the exact mechanisms underlying LBP remain incompletely understood, the destruction of ECM, increased cellular senescence and apoptosis, as well as excessive inflammatory responses, are well-established key drivers of IDD. NPCs are the major cell population that sustains the structural integrity and physiological function of the intervertebral disc. Excessive apoptosis of NPCs is a major pathological basis for IDD, while inflammation and oxidative reactions play crucial roles in NPC apoptosis [13–15]. Therefore, enhancing antioxidant activity and reducing inflammation can effectively inhibit NPC apoptosis, thereby alleviating symptoms of IDD.

With the improvement of living standards and health awareness, people are increasingly focused on developing efficient and low-side-effect drugs from natural resources. In recent years, compounds extracted from natural plants have emerged as promising agents for degenerative disorders, with good therapeutic effects and fewer side effects [16]. As a natural flavonoid, BA is isolated from the roots of the *Scutellaria baicalensis*, appearing as a light yellow powder. Relevant studies have shown that BA exerts diverse pharmacological activities, such as anti-inflammatory, anti-apoptotic, antioxidant, antiviral and cardioprotective. Current research has found that BA is a promising candidate for the clinical intervention of IDD [17–19]. Meanwhile, this study found that BA simultaneously reversed a series of degenerative phenotypes induced by IL-1β. Specifically, BA suppressed abnormal apoptosis in NPCs by elevating anti-apoptotic Bcl-2, reducing pro-apoptotic Bax and blocking c-caspase-3 activation, which is consistent with previously reported anti-apoptotic effects of BA in other cellular models. In terms of inflammation, BA significantly reduced the levels of TNF-α and IL-6. More importantly, this study elucidated the regulatory role of BA in the imbalance in ECM metabolism of IDD. IL-1β disrupts ECM homeostasis, manifested as the upregulation of the catabolic enzyme MMP-3 and the downregulation of anabolic key components Aggrecan and Collagen II. BA treatment effectively reversed this imbalance by suppressing MMP-3 expression and promoting the synthesis of matrix components. Maintaining ECM integrity is critical for delaying IDD development, so this regulatory effect endows BA with great therapeutic potential against disc degeneration. However, the specific mechanisms behind its protective actions against IDD remain unclear and require further investigation.

Network pharmacology is a newly developed interdisciplinary discipline [20]. This systematic methodology constructs interaction networks linking drugs, active components, molecular targets and diseases, to comprehensively explore drug therapeutic mechanisms [21]. It also offers novel perspectives for disease treatment with pharmaceutical agents. In this study, the Cytoscape software was used to reconstruct the PPI network of common genes obtained from the STRING database and identified core genes (AKT1, TP53, MAPK3, CASP3 and TNF, etc.) of BA against IDD. These core target participate in diverse signaling cascades associated with inflammation, apoptosis, cell senescence, metabolism and cell proliferation, etc. GO and KEGG enrichment analysis revealed showed that the therapeutic effects of BA are closely correlated with multiple pathways, including AGE-RAGE, MAPK, TNF, IL-17, PI3K-Akt, apoptosis pathway, etc. Prior studies have confirmed the vital contributions of these pathways to IDD development. Accumulated AGE accumulation activates the AGE/RAGE axis, triggering oxidative stress and endochondral ossification, and further disturbing the microenvironment of NP tissue and further disturbing the osteogenic differentiation of disc cells [22]. The MAPK pathway is a key regulator during IDD pathogenesis. Its activation facilitates inflammatory apoptosis, pro-inflammatory cytokines secretion and upregulation of matrix-degrading enzyme [23, 24]. TNF and IL-17 signaling act synergistically to exacerbate IDD via enhancing inflammatory mediator secretion, inducing NPC apoptosis and accelerating ECM breakdown [14, 25]. Additionally, the PI3K-Akt pathway exerts protective effects against IDD, which is achieved by alleviating ECM degradation, suppressing cell apoptosis and modulating autophagy activity.

Network pharmacological predictions suggested that the MAPK pathway was identified as a key mediator for BA to intervene in IDD. Therefore, this study utilized an IL-1β-induced in vitro model of IDD for validation. The results showed that IL-1β stimulation markedly activated ERK and p38 phosphorylation in NPCs, while BA pretreatment markedly inhibited this activation, suggesting that BA inhibited the activation of the MAPK pathway. Notably, follow-up anisomycin rescue experiments further solidified the MAPK-dependent mechanism underlying BA’s protection. As a canonical MAPK agonist, anisomycin restored the phosphorylation of ERK and p38 suppressed by BA and partially offset BA’s protective effects. Specifically, anisomycin reversed BA-mediated improvements in cell viability and proliferation, facilitated NPC apoptosis, boosted pro-inflammatory cytokine secretion and disrupted ECM homeostasis. Collectively, these rescue findings robustly confirm that BA alleviates IL-1β-triggered inflammation, apoptosis and ECM degradation in NPCs predominantly via inhibition of the MAPK signaling cascade. This study systematically linked the inhibitory effect of BA on the MAPK pathway to its protective role in ECM metabolism, providing a clearer pathway to explain its pharmacological mechanism in an IL-1β-induced NPCs degeneration model.

Several limitations exist in this study. Firstly, all conclusions are drawn from in *vitro* cell experiments at the cellular level, which do not fully replicate the complex mechanical and nutritional microenvironment of the intervertebral disc in *vivo*. Secondly, the predicted molecular targets and signaling cascades have not been comprehensively verified. This work primarily explored the MAPK pathway and additional experiments are required to validate other predicted targets and pathways in subsequent research. Finally, determining the optimal dosage, timing, and evaluating the efficacy and safety of BA in animal models are core issues to be addressed in future translational research.

## Conclusions

In summary, by integrating network predictions with experimental validation, this study confirms that BA exerts a protective effect against IDD via suppressing the MAPK pathway, thereby alleviating inflammation, apoptosis, and ECM degradation in NPCs. The present results clarify the pharmacological mechanisms of BA and lay a preclinical foundation for its clinical translation in IDD therapy.

## Supplementary Information

Below is the link to the electronic supplementary material.

Supplementary Material 1

## Data Availability

The original manuscript contained in the research report is included in the article/Supplementary Materials. Further inquiries can be made directly to the corresponding author.
